# Atrial fibrillation detection in outpatient electrocardiogram monitoring: An algorithmic crowdsourcing approach

**DOI:** 10.1371/journal.pone.0259916

**Published:** 2021-11-16

**Authors:** Ali Bahrami Rad, Conner Galloway, Daniel Treiman, Joel Xue, Qiao Li, Reza Sameni, Dave Albert, Gari D. Clifford

**Affiliations:** 1 Department of Biomedical Informatics, Emory University, Atlanta, GA, United States of America; 2 AliveCor Inc., Mountain View, CA, United States of America; 3 Department of Biomedical Engineering, Georgia Institute of Technology, Atlanta, GA, United States of America; Valahia University of Targoviste: Universitatea Valahia din Targoviste, ROMANIA

## Abstract

**Background:**

Atrial fibrillation (AFib) is the most common cardiac arrhythmia associated with stroke, blood clots, heart failure, coronary artery disease, and/or death. Multiple methods have been proposed for AFib detection, with varying performances, but no single approach appears to be optimal. We hypothesized that each state-of-the-art algorithm is appropriate for different subsets of patients and provides some independent information. Therefore, a set of suitably chosen algorithms, combined in a weighted voting framework, will provide a superior performance to any single algorithm.

**Methods:**

We investigate and modify 38 state-of-the-art AFib classification algorithms for a single-lead ambulatory electrocardiogram (ECG) monitoring device. All algorithms are ranked using a random forest classifier and an expert-labeled training dataset of 2,532 recordings. The seven top-ranked algorithms are combined by using an optimized weighting approach.

**Results:**

The proposed fusion algorithm, when validated on a separate test dataset consisting of 4,644 recordings, resulted in an area under the receiver operating characteristic (ROC) curve of 0.99. The sensitivity, specificity, positive-predictive-value (PPV), negative-predictive-value (NPV), and F1-score of the proposed algorithm were 0.93, 0.97, 0.87, 0.99, and 0.90, respectively, which were all superior to any single algorithm or any previously published.

**Conclusion:**

This study demonstrates how a set of well-chosen independent algorithms and a voting mechanism to fuse the outputs of the algorithms, outperforms any single state-of-the-art algorithm for AFib detection. The proposed framework is a case study for the general notion of crowdsourcing between open-source algorithms in healthcare applications. The extension of this framework to similar applications may significantly save time, effort, and resources, by combining readily existing algorithms. It is also a step toward the democratization of artificial intelligence and its application in healthcare.

## Introduction

Atrial fibrillation (AFib) is the most common cardiac arrhythmia, with an increasing prevalence worldwide [1]. As of 2020, the worldwide prevalence of AFib was 37,574 million cases (0.51% of the worldwide population), and has increased by 33% during the last 20 years [2]. AFib is associated with a five-fold increase in likelihood of ischemic stroke, blood clots, heart failure, coronary artery disease, and two-fold increase in likelihood of death [3]. Despite its prevalence and the importance of its diagnosis, AFib remains under-diagnosed [4], mainly due to asymptomatic individuals and the fact that the electrocardiogram (ECG) is not routinely monitored in everyday life. A recent study estimated that 13.1% of AFib cases were undiagnosed and over half of these were at moderate to high risk of stroke [5]. The ECG is in fact the primary and most accurate means of screening subjects for AFib.

Motivated by the aforementioned factors, AliveCor (Mountain View, CA) developed a low-cost patient-driven personal ECG device (*Kardia*), with a focus on outpatient monitoring (primarily) for AFib detection. Personal ECG devices such as the AliveCor Kardia and the Apple Watch enable the detection on a much larger scale than ever before, currently generating 100,000’s of ECGs a day. However, this places additional burden on healthcare systems, already swamped by systems designed to be extremely sensitive, but not specific. Optimizing the identification of AFib for such personal devices to reduce this burden was the motivation behind the 2017 PhysioNet/Computing in Cardiology (CinC) Challenge and this current research, on which we build in this work.

Many research groups worldwide have worked on ECG classification for AFib detection and other arrhythmias. However, most of these algorithms are evaluated on very limited datasets and compared with only a few other algorithms. This results in algorithms that underperform on larger datasets and/or tend to be more sensitive or more specific on unseen test data. In this work, we develop a new AFib detection algorithm based on existing algorithms and demonstrate that an appropriate fusion mechanism leads to an algorithm with superior performance and highly robust on unseen datasets.

Although the superior performance and robustness of ensemble algorithms is well-known in the field of machine learning [6], the approaches are generally ensembles of weak learners. In this work, 38 different algorithms trained in different conditions independently by independent research teams (i.e., an ensemble of strong learners) are combined to address the issue of detecting AFib from a single ECG channel. To do so, a total of 38 algorithms were investigated: a total of 36 independently developed algorithms sourced from the 2017 PhysioNet/CinC Challenge [7], plus two independently developed algorithms by the coauthors of this paper (AliveCor Inc and Li *et al*. [8]).

The 2017 PhysioNet/CinC Challenge focused on differentiating AFib from noise, non-AFib normal (Normal), and non-AFib abnormal (Other) rhythms in short (9–61 s) single lead-I ECG recordings captured by the AliveCor Kardia device [7]. All PhysioNet/CinC Challenge algorithms were trained on the PhysioNet/CinC training dataset consisting of 8,528 ECG recordings. We were not involved in the training process of these algorithms except Zabihi *et al*. [9], but only had access to the trained algorithms. We emphasize that in this work, the processing steps inside each algorithm, such as preprocessing, noise removal, QRS detection, and classification method, are not important, and we need only the output of each algorithm. The ranking and voting system removes highly flawed algorithms. As the focus of this work is on AFib vs. non-AFib detection, the algorithms were modified slightly such that in the case of Normal and Other rhythms, they generate non-AFib labels (through the fusion of Normal and Other labels produced by each algorithm).

The independently developed algorithm by Li *et al*. [8] was designed to classify AFib from non-AFib using a support vector machine [10] (SVM) model and 14 features extracted from the beat-to-beat interval (RR interval) time-series. The extracted features were from different domains: 7 time-domain, 3 frequency-domain, and 4 nonlinear parameters. A recursive feature elimination algorithm was adopted to select a set of 8 features for classification. The model was trained on the MIT-BIH Atrial Fibrillation Database [11, 12] with a total number of 23 10-hour long ECG records by a ten-fold cross-validation approach.

The AliveCor Kardia algorithm combines ten different processing steps into an SVM for determining a final AFib call. The Kardia algorithm is specifically adapted to processing short, 30 s recordings from AliveCor’s handheld device. First, a deep neural network [13] (DNN)-based method is used to locate and classify QRS complexes into one of three classes: normal, premature ventricular complex (PVC), and premature atrial complex (PAC). After this, an average beat is derived, as well as various classical RR variability metrics. Two additional DNNs are then utilized: a deep convolutional neural network (CNN) that processes the average beat, and a recurrent neural network (RNN) that processes the normalized RR time-series by taking into account beat labels. These two additional DNNs each produce a softmax output. The inputs of the final SVM classifier are the softmax outputs of DNNs and eight classical RR variability features. The AliveCor Kardia algorithm was trained over a total number of 24,000 non-public ECG records.

## Materials and methods

### ECG dataset and data annotation

The training and testing datasets of this study are independent of the three datasets mentioned earlier and both extracted from larger datasets developed in AliveCor Inc. In what follows, we briefly describe the developments of these datasets. 1,589 ECG recordings from unique subjects were selected from the original single-lead Kardia Mobile & Kardia Band validation set, AliveDB1. AliveDB2 was built upon AliveDB1 by adding a set of 1,000 6L-lead recordings each from a unique 6L user, resulting in 2,589 recordings. These recordings were annotated independently by two cardiologists. A third tie-breaker cardiologist labeled the 124 recordings for which the previous annotators disagreed. The labels that annotators provided for the recordings are: inverted (when the polarity of ECG was inverted), sinus rhythm, atrial fibrillation, atrial flutter, bradycardia, tachycardia, wide QRS (QRS ≥ 120 ms), no P-wave, unclassified (when the cardiologist could not classify the rhythm), and unreadable or noisy (when the cardiologist could not read the recording due to a large amount of noise). The training set of this study is a subset of AliveDB2 by removing a set of 57 unsuitable recordings (e.g., pacemaker signals) selected by a cardiologist. In addition, in this study, we only use the Lead I signal. The final training set has 2,532 30 s single-lead ECG recordings. We limited the number of classes to three: AFib, noisy, and non-AFib (rhythms that are neither AFib nor noisy). The distribution of these classes in the training dataset is as follows: 2,317 non-AFib, 137 AFib, and 78 noisy.

The 6L-2020 dataset is composed of a set of 18,850 2-lead recordings from unique users made with Kardia 6L. This dataset is not fully randomized such that it is sampled specifically to increase the prevalence of arrhythmias and unusual heart rates. In this dataset, the ECG recordings were divided into blocks of 1,000 and annotated by CardiacMinds, using AliveCor’s annotation tool. Each block of 1,000 contained 940 unique files and 60 duplicate files, except for Block 1, which contained 990 unique files and only 10 duplicates. Duplicates were added with modified unique IDs and used to ensure consistency between multiple annotators. After annotation by CardiacMinds, a cardiologist reviewed the first 5 blocks (5,000 recordings) and corrected the annotations. Then, the 250 duplicate records were removed from this 5-block data to obtain 4,750 unique recordings. The existing labels in this dataset are: normal sinus rhythm, sinus arrhythmia, atrial fibrillation, atrial flutter, first degree AV block, second degree AV block (type I), second degree AV block (type II), third-degree AV block, accelerated junctional rhythm, atrial bigeminy, atrial trigeminy, atrial high order ectopy (>trigeminy), ventricular bigeminy, ventricular trigeminy, ventricular high order ectopy (> trigeminy), supraventricular tachycardia (SVT), SVT run, nonsustained ventricular tachycardia, ventricular tachycardia, wide QRS, unreadable, and inverted. Some recordings do not have any labels, meaning that the cardiologist has not decided to allocate any label to them due to uncertainty. The testing dataset of this study is the remaining 4,644 recordings after removing all unlabeled, noisy, and unsuitable records from the 5-block data. In the testing dataset, we only use Lead I data and two class labels: AFib and non-AFib, which encompasses all class labels except AFib, noisy, and unlabeled. The testing dataset consisted of 777 AFib and 3,867 non-AFib.

### Ranking and selecting the algorithms/features

A total of 38 recently developed AFib detection algorithms were studied. The studied algorithms involved various machine learning methods, including but not limited to bagged decision trees [14], random forests [15], deep neural networks, and SVM. We aimed to design a fusion mechanism to combine these base-level algorithms to increase the performance of automatic AFib detection. To add some ECG contextual information [16] to our algorithm, we also provided a set of 24 ECG features derived from Li *et al*. [8] (*n* = 14) and Kardia (*n* = 10). The combination of the outputs of 38 base-level learners (algorithms) and the 24 features was treated as a new set of features (*n* = 62) for a meta-level learner.

The 14 features employed by Li *et al*. [8] are mRR (mean of RR intervals), minRR (minimum of RR intervals), maxRR (maximum of RR intervals), medHR (median of heart rate), SDNN (standard deviation of RR intervals), PNN50 (percentage of RR intervals larger than 50 ms), RMSSD (square root of the mean squared differences of successive RR intervals), LF (low-frequency power), HF (high-frequency power), LF/HF (the ratio of LF to HF), COSEn1 (coefficient of sample entropy), NFEn (normalized fuzzy entropy), MAD (median of the variation in the absolute standard deviation from the mean of heart rate in three adjacent RR segments), and AFEv (an AF evidence feature, as a numeric representation of the Lorenz plot).

The 10 features employed by the Kardia algorithm are RMSSD1 (square root of the mean squared differences of successive RR intervals), RMSSD2 (square root of the mean squared differences of every other RR interval), RMSSD3 (square root of the mean squared differences of every third RR interval), RMeSSD1 (square root of the median squared differences of successive RR intervals), RMeSSD2 (square root of the median squared differences of every other RR interval), RMeSSD3 (square root of the median squared differences of every third RR interval), mHR (mean heart rate), COSEn2 (coefficient of sample entropy), CNNout (output of CNN that processes average beat), and RNNout (output of RNN that processes RR time-series). These 10 features are calculated after a deep neural network algorithm locates and classifies QRS complexes into either normal, PAC, or PVC. It is noteworthy that although some feature types are the same between Kardia and those used by Li *et al*. [8], their values are different because the algorithms use different QRS detection methods. Li *et al*. [8] combine three widely used QRS detection algorithms with the majority voting of the results to calculate the RR intervals.

To train the meta-level learner (i.e., fusing algorithm), we first selected the most relevant and informative meta-level features (i.e., AFib detection algorithms and ECG features) by ranking them using a random forest classifier and randomizing (permuting) the values of each feature. The algorithms/features were sorted in the reverse order based on their out-of-bag errors; the higher the error, the better the algorithm/feature. The rationale is that by permuting or randomizing the output values of an algorithm or the values of a feature, if the random forest classifier’s performance drops, the contribution of that algorithm/feature is significant. But, if randomizing the values of an algorithm or a feature does not have any significant effect on the performance of the classifier (a small out-of-bag error), it indicates that the algorithm/feature is not important for classification and can be removed without a significant drop in the performance. This is a well-known approach based on a built-in characteristic of the random forest classifier for feature selection [15, 17].

### Fusion mechanism

Various fusion mechanisms or voting strategies are proposed in the literature ranging from naïve voting system based on the best performance of algorithms, to LASSO, LASSO+ [16], and Bayesian approaches [18–21], to combine the algorithms’ outputs in smarter and systematic ways. In this work, we examined multiple bagged and boosted decision trees to combine different algorithms, including bagged decision tree, random forest, AdaBoost [22], RUSBoost [23], and TotalBoost [24]. After testing each approach in a repeated (five times) stratified ten-fold cross-validation architecture on the training dataset [25], we conclude that the result of the random forest model was both better and more robust (i.e., varied less among different folds of the cross-validation procedure), as compared with the other methods that we evaluated. It is worth mentioning that due to the size of our training dataset (2,532), we did not try data-hungry algorithms such as deep neural networks.

A random forest classifier is a type of bagged decision tree that samples the features in each training step. In this case, it generates an ensemble of nearly uncorrelated predictors (decision trees) [26]. The training of a random forest classifier involves bagging, i.e., multiple sampling of the training data using the bootstrap method. We sampled the original data with replacement until the subset had the same size as the original data. The decision tree was then trained on that subset and expanded (grown) by finding the best split (based on the cross-entropy) among a randomly selected set of features. We let the trees grow deep (i.e., until each terminal node has only one unique data sample). In growing the trees for classification, a square root of features is typically randomly sampled in each node. We did the same for the first random forest classifier used for algorithm ranking. However, we set the number of (randomly sampled) features to unity to guarantee that the ensemble of predictors became uncorrelated for training the fusing random forest. This number was also cross-validated on the training dataset. In this way, 500 predictors were generated, which at the end, the decision is made by collecting their votes and choosing the candidate (hypothesis) that received the majority of votes.

In this work, the votes (decisions) of only seven top-ranked algorithms (cf. the next section) are collected and given to each predictor as input (feature vector). Each of the seven algorithms classifies each test sample and generates the first-level outputs (votes). Six of the votes are categorical (class labels); however, Kardia’s vote is in the form of a class-specific continuous output. Then, each of the 500 predictors (decision trees) classifies the feature vectors associated with the test samples and generates the final 500 votes. The final decision is made by choosing the hypothesis (class label) that received the majority of votes. It can also be in the form of a class-specific continuous output (e.g., probability) as a ratio of votes allocated to each class.

## Results

### Sorted algorithms/features

Table 1 shows the rank of each algorithm/feature on the training dataset along with the performance of each algorithm. The noisy labels were removed from the training dataset and only used AFib and non-AFib rhythms for ranking the algorithms. The same procedure was applied for assessing the performance of Kardia and Li *et al*. [8] algorithms. However, for evaluating the performance of 36 PhysioNet/CinC algorithms, we used all three classes but at the end, considered non-AFib and noisy labels as the negative class. The reason for this modification is that Kardia and Li *et al*. [8] algorithms are binary classifiers, but the modified versions of PhysioNet/CinC algorithms are 3-class classifiers.

**Table 1:** The 38 base-level algorithms and the 24 ECG features were ranked using a random forest classifier. The detailed descriptions of the features are described in Methods. The classification results of the 38 algorithms on the training dataset are also listed. For features the corresponding locations of classification results are filled with N/A (i.e., not applicable). The source codes and their related papers can be found in https://physionetchallenges.org/2017/results/.

Rank	Algorithm/Feature	Entry Code	Sensitivity	Specificity	PPV	NPV	F1-score	AUC
1	Kardia	—–	0.905	0.999	0.976	0.994	0.939	0.999
2	Datta *et al*. [27, 28]	shreyasi-datta-209	0.898	0.993	0.872	0.994	0.885	0.945
3	Baydoun *et al*.	mohammed-baydoun-208	0.898	0.996	0.932	0.994	0.915	0.947
4	Kropf *et al*. [29, 30]	martin-kropf-205	0.920	0.995	0.907	0.995	0.913	0.957
5	Zabihi *et al*. [9]	morteza-zabihi-208	0.883	0.996	0.931	0.993	0.906	0.940
6	Gliner *et al*. [31, 32]	gliner-vadim-210	0.869	0.995	0.908	0.993	0.888	0.932
7	Soliński *et al*. [33]	rymko-207	0.883	0.989	0.823	0.993	0.852	0.936
8	Patidar *et al*. [34]	ashish-sharma-210	0.891	0.994	0.891	0.994	0.891	0.942
9	Jiménez-Serrano *et al*. [35]	elena-simarro-mondejar-216	0.832	0.996	0.919	0.990	0.874	0.914
10	Sopic *et al*. [36]	dionisije-sopic-208	0.854	0.995	0.907	0.992	0.880	0.925
11	Liu *et al*. [37]	na-liu-210	0.869	0.993	0.882	0.993	0.875	0.931
12	Sadr *et al*. [38]	nadi-sadr-208	0.891	0.972	0.649	0.994	0.751	0.932
13	Yazdani *et al*. [39]	sasan-yazdani-204	0.818	0.995	0.903	0.990	0.858	0.906
14	CNNout	—–	N/A	N/A	N/A	N/A	N/A	N/A
15	Li *et al*. [8]	—–	0.978	0.975	0.698	0.999	0.815	0.977
16	AFEv [8]	—–	N/A	N/A	N/A	N/A	N/A	N/A
17	Jiayu *et al*.	chen-jiayu-202	0.854	0.993	0.867	0.992	0.860	0.923
18	minRR [8]	—–	N/A	N/A	N/A	N/A	N/A	N/A
19	Ocoa	victor-manuel-jose-ocoa-202	0.876	0.990	0.828	0.993	0.851	0.933
20	Plesinger *et al*. [40, 41]	filip-plesinger-210	0.832	0.993	0.877	0.990	0.854	0.913
21	NFEn [8]	—–	N/A	N/A	N/A	N/A	N/A	N/A
22	Christov *et al*. [42, 43]	ivaylo-christov-204	0.890	0.989	0.824	0.994	0.856	0.940
23	MAD [8]	—–	N/A	N/A	N/A	N/A	N/A	N/A
24	Stepien *et al*. [44]	katarzyna-stepien-209	0.839	0.993	0.865	0.991	0.852	0.916
25	Mahajan *et al*. [45]	oguz-akbilgic-219	0.781	0.996	0.922	0.988	0.846	0.889
26	Costa *et al*.	javier-de-la-torre-costa-205	0.825	0.993	0.863	0.990	0.843	0.909
27	RNNout	—–	N/A	N/A	N/A	N/A	N/A	N/A
28	LFn [8]	—–	N/A	N/A	N/A	N/A	N/A	N/A
29	Mei *et al*.	zhenning-mei-209	0.796	0.988	0.784	0.988	0.790	0.892
30	Chandra *et al*. [46]	b-s-chandra-207	0.883	0.977	0.688	0.993	0.773	0.930
31	RMSSD2	—–	N/A	N/A	N/A	N/A	N/A	N/A
32	RMeSSD1	—–	N/A	N/A	N/A	N/A	N/A	N/A
33	Liu *et al*. [47]	runnan-he-210	0.825	0.985	0.753	0.990	0.788	0.905
34	COSEn1 [8]	—–	N/A	N/A	N/A	N/A	N/A	N/A
35	RMeSSD2	—–	N/A	N/A	N/A	N/A	N/A	N/A
36	mRR [8]	—–	N/A	N/A	N/A	N/A	N/A	N/A
37	Bonizzi *et al*. [48]	joel-karel-203	0.869	0.969	0.617	0.992	0.721	0.919
38	mHR	—–	N/A	N/A	N/A	N/A	N/A	N/A
39	Da Silva-Filarder *et al*. [49]	matthieu-da-silva-filarder-204	0.839	0.976	0.665	0.991	0.742	0.908
40	RMSSD1	—–	N/A	N/A	N/A	N/A	N/A	N/A
41	COSEn2	—–	N/A	N/A	N/A	N/A	N/A	N/A
42	HFn [8]	—–	N/A	N/A	N/A	N/A	N/A	N/A
43	RMSSD3	—–	N/A	N/A	N/A	N/A	N/A	N/A
44	medHR [8]	—–	N/A	N/A	N/A	N/A	N/A	N/A
45	LF/HF [8]	—–	N/A	N/A	N/A	N/A	N/A	N/A
46	Ghiasi *et al*. [50]	kamran-kiani-203	0.730	0.995	0.885	0.985	0.800	0.862
47	SDNN [8]	—–	N/A	N/A	N/A	N/A	N/A	N/A
48	RMeSSD3	—–	N/A	N/A	N/A	N/A	N/A	N/A
49	Ferrís *et al*.	lluis-borras-ferris-205	0.730	0.982	0.694	0.984	0.712	0.856
50	Marques *et al*.	ines-chavarria-marques-204	0.336	0.997	0.868	0.963	0.484	0.666
51	RMSSD [8]	—–	N/A	N/A	N/A	N/A	N/A	N/A
52	Goovaerts *et al*.	griet-goovaerts-206	0.620	0.993	0.842	0.979	0.714	0.807
53	Hasna *et al*. [51]	octavian-lucian-hasna-202	0.256	0.973	0.350	0.958	0.295	0.614
54	Álvarez *et al*.	pedro-alvarez-204	0.810	0.986	0.766	0.989	0.787	0.898
55	PNN50 [8]	—–	N/A	N/A	N/A	N/A	N/A	N/A
56	Santos *et al*.	carlos-fambuena-santos-202	0.175	0.972	0.264	0.954	0.211	0.574
57	Hassanat *et al*.	ahmad-hassanat-206	0.679	0.967	0.541	0.981	0.602	0.823
58	Kalidas	vignesh-kalidas-204	0.752	0.980	0.678	0.986	0.713	0.866
59	Amandi *et al*.	ruhallah-amandi-205	0.467	0.966	0.441	0.969	0.454	0.717
60	Wang	ludi-wang-206	0.788	0.986	0.761	0.988	0.774	0.887
61	Jekova *et al*. [52]	irena-jekova-204	0.584	0.970	0.530	0.976	0.556	0.777
62	maxRR [8]	—–	N/A	N/A	N/A	N/A	N/A	N/A

### Fusion of the selected algorithms

After ranking the algorithms/features by the approach mentioned above, a different number of top-ranked algorithms/features were selected and fed into another random forest classifier for AFib detection. By performing a grid search in a repeated (five times) stratified ten-fold cross-validation procedure for algorithm/feature selection and hyperparameter tuning [25], it was concluded that selecting more than seven top-ranked algorithms/features would not improve the classification performance in terms of the F1-score on the training dataset. So, in this work, we selected the seven top-ranked algorithms. The outputs of these algorithms were then fused to train another random forest classifier, using the training dataset and the parameters derived from the aforementioned cross-validated method.

**Fig 1:**
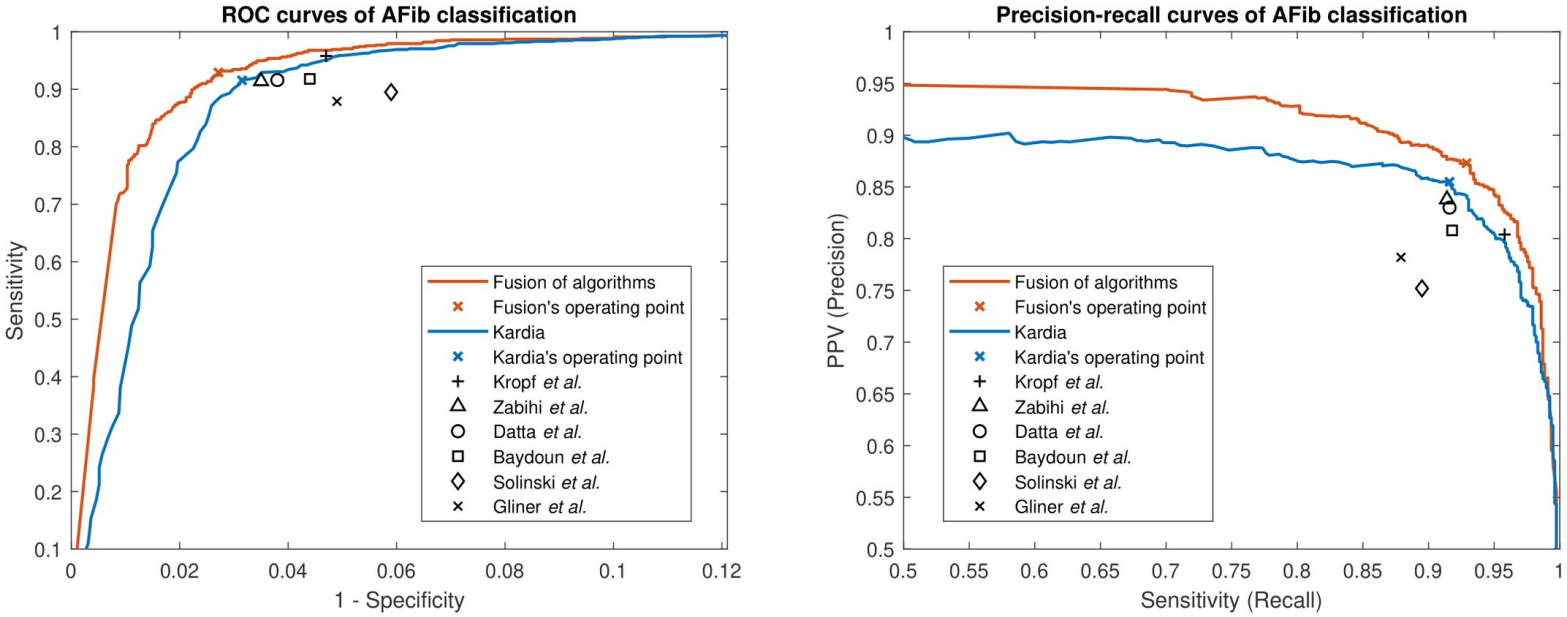
ROC and precision-recall curves. The left panel shows the ROC curves of our proposed algorithm (fusion of algorithms) and Karida. Since the other six algorithms do not generate class-specific continuous output (they generate only a class label), their operating points are indicated with one point defined by their sensitivity and specificity. The right panel shows the precision-recall curves of our proposed algorithm and Kardia along with the operating points of the other six algorithms.

The proposed model’s performance was assessed on the test dataset. The proposed model achieved an area under the receiver operating characteristic (ROC) curve (AUC) of 0.99 for AFib detection. Its sensitivity, specificity, positive-predictive-value (PPV), negative-predictive-value (NPV), and F1-score were 0.93, 0.97, 0.87, 0.99, and 0.90, respectively. Fig 1 shows the ROC and precision-recall curves of the fusion of algorithms and the performance of individual top-ranked algorithms participating in the fusion algorithm. As can be seen, the fusion of algorithms surpasses each individual algorithm’s performance.

Table 2 shows the performance of the fusion algorithm as well as the performance of selected algorithms using the aforementioned algorithms/features ranking strategy and the cross-validation procedure. From this table, it is seen that the performance of the fusion of algorithms is superior to any individual algorithm across all metrics tested. It is noteworthy that for evaluating the performance, one needs to consider all criteria together. For example, although the algorithm by Kropf *et al*. [29] has the highest sensitivity and NPV, its overall performance is not the highest when considering all performance criteria. This can be observed in Fig 1 by the distance of the operating point of Kropf’s algorithm from the ideal points (i.e., upper left corner in the ROC curve figure and upper right corner in the precision-recall curve figure).

**Table 2:** The classification results of the selected algorithms as well as the fusion algorithm on the test dataset.

Algorithm	Sensitivity	Specificity	PPV	NPV	F1-score	AUC
Kardia	0.916	0.969	0.855	0.983	0.885	0.983
Kropf *et al*. [29]	0.958	0.953	0.804	0.991	0.874	0.955
Zabihi *et al*. [9]	0.914	0.965	0.838	0.982	0.874	0.939
Datta *et al*. [27]	0.916	0.962	0.830	0.983	0.871	0.939
Baydoun *et al*.	0.918	0.956	0.808	0.983	0.859	0.937
Soliński *et al*. [33]	0.895	0.941	0.752	0.978	0.817	0.918
Gliner *et al*. [31]	0.879	0.951	0.782	0.975	0.827	0.915
Fusion of Algorithms	0.929	0.973	0.873	0.986	0.900	0.988

Table 2 also shows that among the top seven selected meta-level features (i.e., AFib detection algorithms and ECG features), all of them were the output values of the algorithms, and no ECG feature was selected. However, it is noteworthy that at least ten features contributed to the final algorithm through the Kardia algorithm (Kardia was selected in the final set).

Table 2 specifically demonstrated that the proposed method outperforms the Kardia algorithm (i.e., the best single algorithm) by 0.015 and 0.005 in terms of F1-score and AUC, respectively. At first glance, it seems that this improvement is marginal. However, it is better understood in terms of reduced “false positives” and reduced “false negatives”. Considering 100,000 ECG recordings per day and AFib prevalence of 0.5% (Lippi *et al*. [2]), the number of AFib and non-AFib recordings will be 500 and 99,500 per day, respectively. The 2.7% false-positive rate of our proposed method (see next section) leads to 2,687 false-positive errors per day. If we do the same calculation for Kardia algorithms with 3.1% false-positive rates, the number of false-positive errors will be 3,085 per day. This number is 398 cases more than our proposed method and typically require case-by-case screening by human experts. On the other hand, the 7.1% false-negative rate of our proposed method translates to 36 false-negative errors per day (i.e., AFib cases which are not diagnosed). This number for the Kardia algorithm with 8.4% false-negative rates will be 42, which is 6 cases more. It is worth mentioning that although the aforementioned 0.5% is AFib prevalence worldwide, we expect a higher prevalence among the outpatient monitored populations. For example, if we use 2% AFib prevalence reported by Zoni-Berisso *et al*. [53] the reduced false positives and reduced false negatives will be 392 and 26 cases, respectively. Moreover, if we use the 5.4% AFib prevalence in our training dataset then we will have 379 and 71 reduced false positives and reduced false negatives, respectively.

Considering that all other six selected algorithms have significantly inferior performance to Kardia in terms of F1-score and AUC, if we repeat the same experiment by designing a fusion mechanism to combine those six algorithms, we arrive at the same observation. The new model achieves a 0.895 F1-score and 0.980 AUC, respectively. These results are comparable to the Kardia algorithm’s performance. However, the reason that the performance of the fusion of seven algorithms is higher than Kardia is that the other six algorithms provide some independent information from Kardia, and the random forest is able to determine which algorithms to weight more in different circumstances.

### Sources of Misclassification

**Fig 2:**
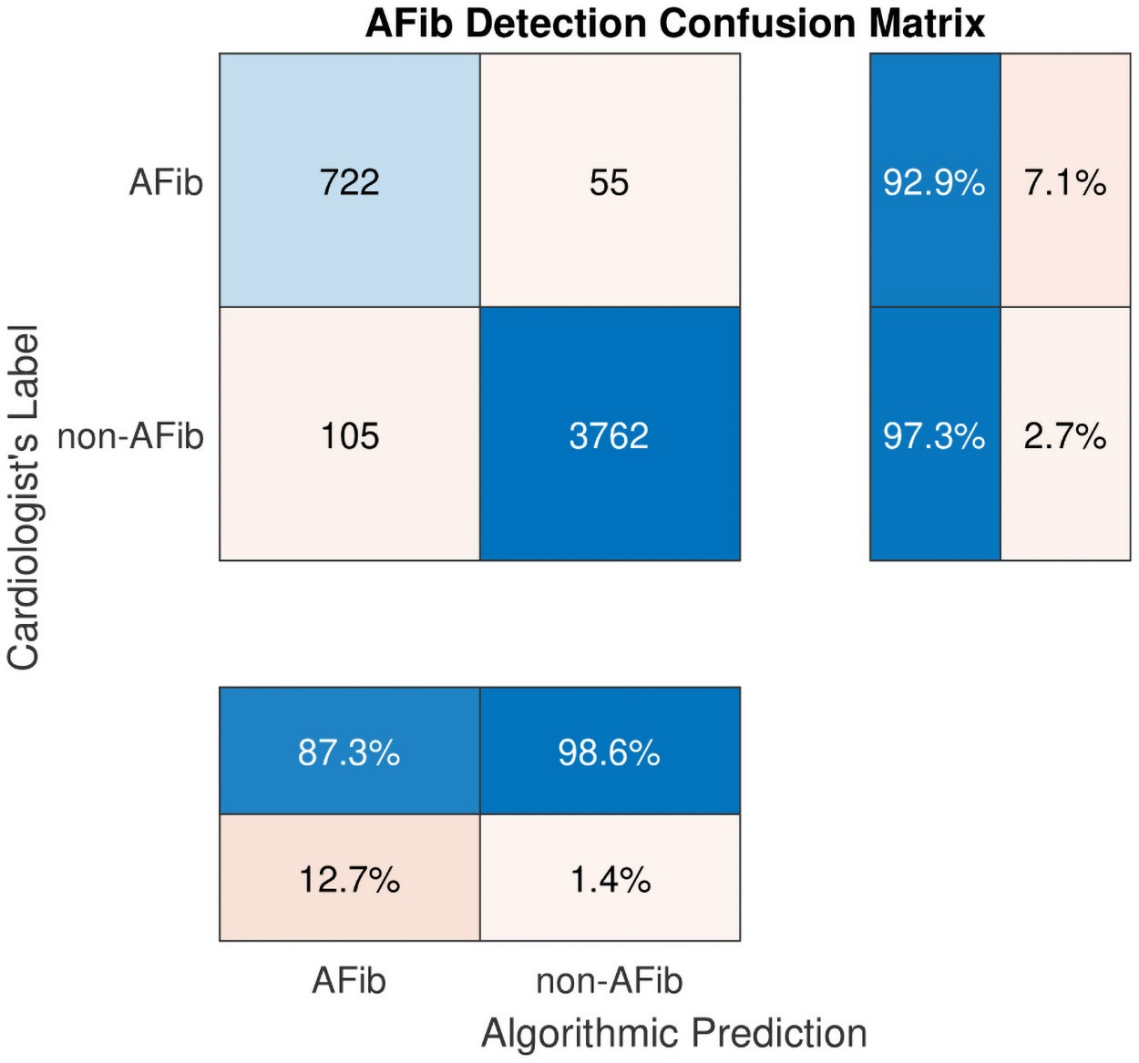
Confusion matrix for the proposed AFib detection algorithm.

**Fig 3:**
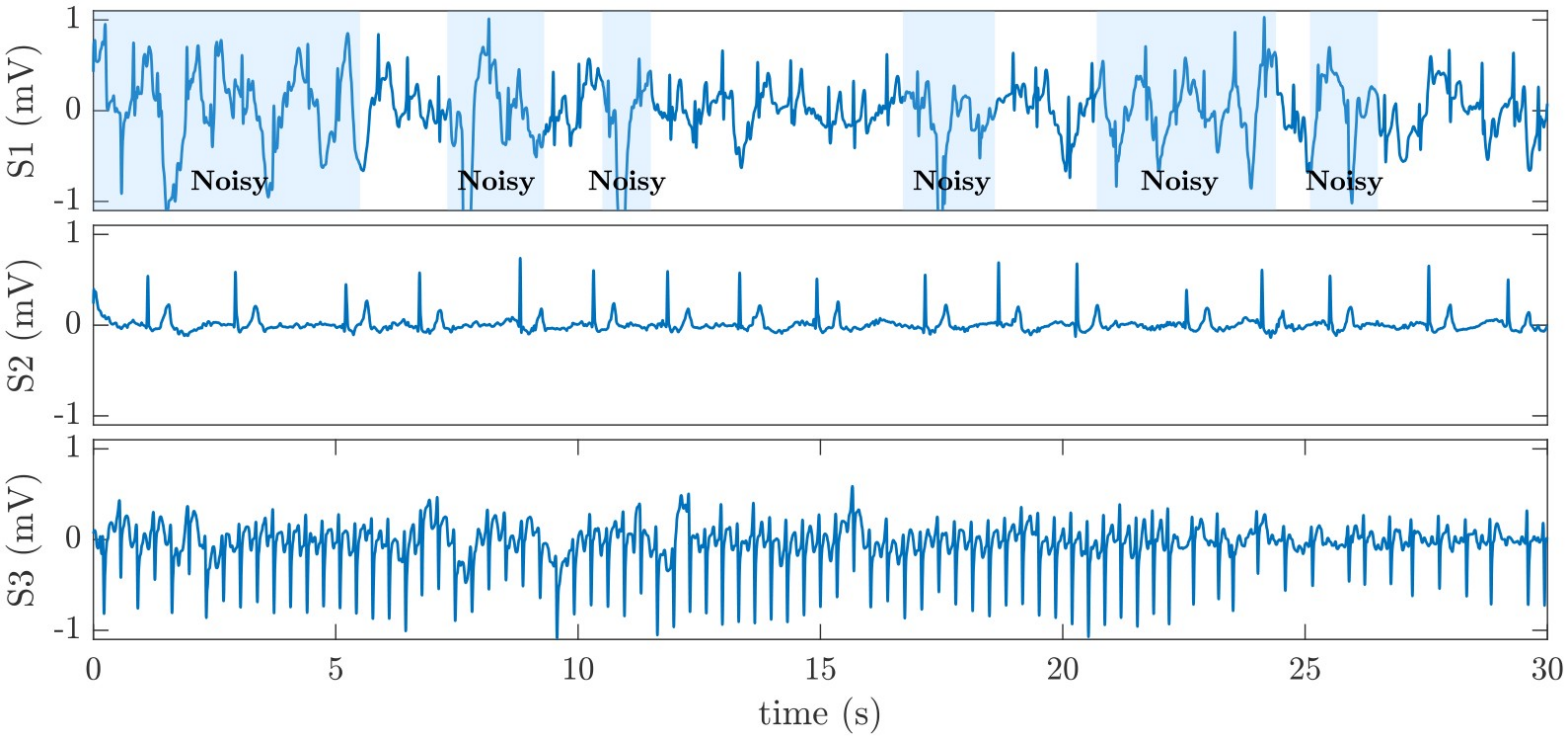
Examples of false-negative errors in AFib detection. S1 is a 30 s noisy ECG segment in the test dataset labeled as AFib but incorrectly classified as non-AFib. S2 is a slow AFib rhythm with a 34 bpm ventricular rate that is classified as non-AFib. S3 is a segment of AFib with supraventricular tachycardia (SVT) that is incorrectly classified as non-AFib.

Fig 2 shows the detailed results of the proposed model in the form of a confusion matrix. Accordingly, from 777 (= 722 + 55) AFib recordings, 722 are detected correctly, resulting in a sensitivity of 92.9%. However, 55 AFib instances are incorrectly classified as non-AFib (false negatives), which results in a 7.1% false-negative rate. Further investigation in the dataset reveals that most false-negative errors belong to those AFib recordings that are either noisy or slow. By slow AFib, we refer to AFib rhythms that have a ventricular rate less than 60 bpm. Moreover, there are also a few instances of AFib/supraventricular tachycardia (SVT) that are incorrectly classified as non-AFib. Fig 3 shows typical instances of these types of false negative errors.

**Fig 4:**
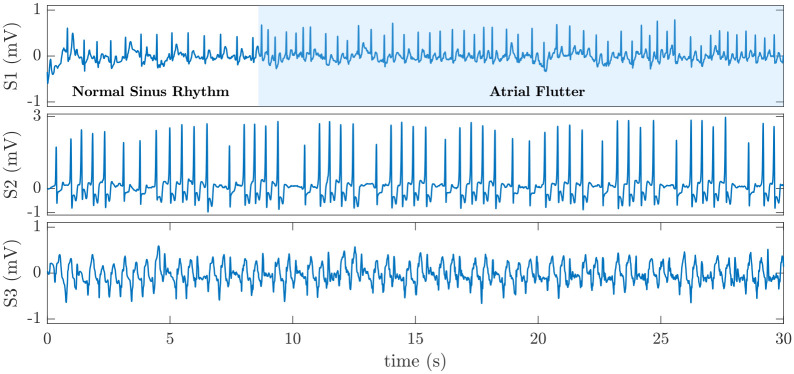
Examples of false-positive errors in AFib detection. S1 is a 30 s ECG signal in which an episode of atrial flutter follows an episode of normal sinus rhythm. This ECG signal is classified as AFib. S2 is an episode of NSVT that is incorrectly classified as AFib. S3 is an episode of SVT with wide QRS that is incorrectly classified as AFib.

The confusion matrix in Fig 2 also shows that among 3,867 (= 3762+ 105) non-AFib recordings, 3,762 are classified as non-AFib, and 105 are classified as AFib resulting in 97.3% specificity and a 2.7% false-positive rate, respectively. Again, a case-by-case visual inspection of the dataset shows that among those 105 non-AFib recordings classified as AFib (false positive error), nearly half of them are atrial flutter. In fact, discrimination between AFib and atrial flutter from only lead I is sometimes difficult. We conjecture that if lead II data were available, it was much easier to differentiate between AFib and atrial flutter. The remaining false-positive errors include nonsustained ventricular tachycardia (NSVT), atrial bigeminy, first-degree AV block, third-degree AV block, SVT, atrial high order ectopy, and sinus arrhythmia with wide QRS (QRS ⩾ 120 ms). Fig 4 shows some instances of false-positive errors in our dataset.

## Discussion

This study designed and demonstrated a fusion-based algorithm for crowdsourcing between open-source state-of-the-art algorithms for AFib classification. The proposed scheme outperformed any individual algorithm across all metrics. This indicated that the fusion mechanism has successfully learned and combined the best aspect of each algorithm. In particular, the importance of having high specificity without sacrificing sensitivity should not be underestimated. With clinical applications involving the screening of large numbers of individuals, confidence in the determination of AFib is highly advantageous for practical implementation. In fact, if there are significant numbers of false positives, then the burden is on the human experts (the healthcare system in the large scale) to adjudicate the interpretations. With the large numbers of readings that can be generated with consumer-focused devices, the burden can be overwhelming.

As reported in our previous work [54], the framework presented here for fusing between independent algorithms developed by different research teams is a generalized approach that could represent a new paradigm for the future of the application of artificial intelligence (AI) in medicine. However, considering machine learning and AI as a panacea for all problems in designing the decision-making systems is dangerous, particularly in healthcare. This is why we deliberately focused on and underlined the instances that our designed algorithm failed to differentiate between AFib and non-AFib rhythms. We also note that our data did not include any information on race, and so we were unable to evaluate the performance of the voting approach in this domain. We note that this is an important area to explore before translating the discoveries into practice.

Any data-driven algorithmic performance depends on the quality and size of the training and test data. In this regard, if a specific type of examples were missing in the training dataset or only a few samples are available, the algorithm cannot learn to differentiate between that class and others (the imbalanced dataset phenomenon). For example, it was already discussed that the developed algorithm incorrectly detects an episode of NSVT as AFib. The main reason for this is that the number of instances of NSVT in our dataset is minimal, so the algorithm cannot effectively learn to classify this rhythm as non-AFib, and mostly focuses on the changes in RR-intervals, thus classifying it as AFib. While expanding the labeled set of data, or include more (independent) algorithms is likely to improve the framework, this cannot solve all the issues. As noted earlier, nearly half of the false-positive errors of our fusion algorithm are due to the classification of atrial flutter rhythms as AFib. It is well-known that flutter waves are observed more easily in lead II compared to lead I. We therefore conclude that one either needs to include lead II data in future analysis, or at least to be aware of this limitation and to clearly report it in any machine-based AFib detection/reporting system, to avoid false alarms.

The relatively small size of the dataset used in this study, and the prevalence of rhythms narrow down our options to algorithms that are less data demanding and sensitive to the class imbalance problem. In fact, the selected fusion algorithm in this work (i.e., a random forest) is a good choice that can handle both the data size and class imbalance limitations well. However, we conjecture that developing a much larger labeled dataset (and with more leads) will help with the selection of a higher number of base algorithms and the combination of their decisions will significantly boost performance. Nevertheless, the study described in this work demonstrates that the combination of algorithms with appropriate voting mechanisms is an effective way for decision-making, specifically in the healthcare domain, in which designing new algorithms is typically very time-consuming and expensive. The role of public data and public competitions to generate these independent algorithms is crucial.

## Supporting information

S1 Table The labels of the test dataset. (CSV)

S2 Table The values of the selected algorithms for the test dataset. These values are the inputs of the fusion algorithm.
(CSV)

S3 Table The output values of the fusion algorithm. (CSV)
